# Mechanisms of Vernalization-Induced Flowering in Legumes

**DOI:** 10.3390/ijms23179889

**Published:** 2022-08-31

**Authors:** Svetlana Yu. Surkova, Maria G. Samsonova

**Affiliations:** Mathematical Biology and Bioinformatics Laboratory, Peter the Great St. Petersburg Polytechnic University, Polytechnicheskaya, 29, 195251 St. Petersburg, Russia

**Keywords:** vernalization response, legumes, flowering time, cold-induced flowering, *FLOWERING LOCUS T (FT)*, *SUPPRESSOR OF OVEREXPRESSION OF CONSTANS 1 (SOC1)*, *FLOWERING LOCUS C (FLC)*

## Abstract

Vernalization is the requirement for exposure to low temperatures to trigger flowering. The best knowledge about the mechanisms of vernalization response has been accumulated for *Arabidopsis* and cereals. In *Arabidopsis thaliana*, vernalization involves an epigenetic silencing of the MADS-box gene *FLOWERING LOCUS C (FLC)*, which is a flowering repressor. *FLC* silencing releases the expression of the main flowering inductor *FLOWERING LOCUS T (FT)*, resulting in a floral transition. Remarkably, no *FLC* homologues have been identified in the vernalization-responsive legumes, and the mechanisms of cold-mediated transition to flowering in these species remain elusive. Nevertheless, legume *FT* genes have been shown to retain the function of the main vernalization signal integrators. Unlike *Arabidopsis*, legumes have three subclades of *FT* genes, which demonstrate distinct patterns of regulation with respect to environmental cues and tissue specificity. This implies complex mechanisms of vernalization signal propagation in the flowering network, that remain largely elusive. Here, for the first time, we summarize the available information on the genetic basis of cold-induced flowering in legumes with a special focus on the role of *FT* genes.

## 1. Introduction

In the winter plant varieties, flowering could be initiated only after prolonged cold exposure or vernalization [1,2,3]. The role of vernalization in plant development is to maintain a vegetative state during winter via the repression of major flowering inductors. This ensures that flowering and sexual reproduction take place in the milder conditions of spring [4]. After the end of the cold treatment, the floral activator genes are de-repressed. This leads to their activation by the photoperiod pathway and floral transition [5].

The best knowledge on the cold-induced flowering has been accumulated for *Arabidopsis* and cereals. In *Arabidopsis thaliana*, the response to vernalization is mostly mediated through the MADS-box gene *FLOWERING LOCUS C (FLC)* [6,7]. *FLC* is a repressor that delays flowering by preventing the expression of floral activators [8]. Vernalization treatment represses *FLC* and releases flowering promotion. It has been shown that the major targets of *FLC* in *Arabidopsis* are *FLOWERING LOCUS T* (*FT*) and *SUPPRESSOR OF OVEREXPRESSION OF CONSTANS 1* (*SOC1*) [9,10].

The *Arabidopsis FT* gene functions as an integrator, converging information from the photoperiodic, vernalization and autonomous regulatory pathways [11]. The interplay between activating and repressive inputs from different pathways is controlled through a complex cis-regulatory region [12]. The homologues of the *FT* gene are functioning in a variety of plant species [13,14,15,16,17,18,19,20,21,22,23].

*FT* encodes a major florigen which promotes transition from vegetative growth to flowering [9,24]. A protein product of the *FT* gene accumulates information on environmental signals in the leaves, and then moves to the shoot apex to transfer this information to meristem identity genes, including the key genes *APETALA1 (AP1)* and *LEAFY (LFY)* [25,26,27,28,29,30]. These genes function to convert meristem to the reproductive state. *FT* activates *LFY* expression through the MADS-domain transcription factor SOC1 [5,31,32]. Moreover, *AP1* and *LFY* act as mutual transcriptional activators [33].

An inductive function of the *FT* gene product is opposed by the *TERMINAL FLOWER 1 (TFL1)* gene, which represses flowering and controls inflorescence architecture via the down-regulation of *AP1* and *LFY* [34,35,36,37].

In legumes, vernalization sensitivity, inherent to wild germplasm, has been mostly lost from cultivated genotypes due to the long-standing breeding efforts aimed at creating the early-flowering spring cultivars. Nevertheless, some cultivated genotypes still retain a low level of vernalization response [38,39]. Understanding the genetic bases of cold-induced flowering in legumes enables one to decipher the origin of adaptation to different environments, history of domestication, and evolution of flowering time networks. However, the molecular mechanisms of vernalization response in legumes remain largely obscure.

Remarkably, the *FLC* gene is missing in many legume species. This comprises all galegoid legumes, including *Medicago truncatula*, *Pisum sativum*, *Vicia faba*, *Lens culinaris* and *Cicer arietinum*, and genistoid legumes such as *Lupinus angustifolius*, *Lupinus luteus* and *Lupinus albus* [40,41]. Despite the absence of the *FLC* gene, these species possess a vernalization-based flowering promotion through the activation of *FT* genes (Table 1) [42,43]. The published results suggest that *FT* genes are the main targets of vernalization in legumes (Table 2), although the mechanisms of *FT* activation are still unclear [44,45]. So far, the most intensive research of cold-induced flowering has been conducted in *Medicago trancatula* and narrow-leafed lupin *Lupinus angustifolius* [44,45,46,47]. Here, we summarize the available information for genetic bases of vernalization in eight legume species with a special emphasis on the role of *FT* genes.

## 2. The Mechanism of Cold–Induced Flowering in *Arabidopsis*

Prior to vernalization, flowering promotion in *Arabidopsis* is repressed by the MADS-box transcription factor FLC, which interacts with the first intron of the *FT* gene and the promoter region of *SOC1* gene [8]. FLC represses *FT* expression in the leaves, while *SOC1* is down-regulated in the shoot apex [8,11].

The direct targets of FLC include *SHORT VEGETATIVE PHASE (SVP)* [59], *TEMPRANILLO 1 (TEM1)* [60,61], *SQUAMOSA PROMOTER-BINDING-LIKE PROTEIN 15 (SPL15)* [62,63], and a few more flowering-related genes (summarized in [64]).

*FLC* expression is up-regulated by the *FRIGIDA (FRI)* gene through a FRI-containing supercomplex, which establishes a local chromosomal environment for a high-level production of *FLC* mRNAs [65,66].

The vernalization treatment leads to the epigenetic silencing of *FLC*, which involves multiple factors. These factors include (1) the components of autonomous pathway; (2) the long noncoding RNAs (lncRNAs) produced by *FLC* locus (COOLAIR complex); (3) Polycomb repressive complex 2 (PRC2), including the core components VERNALIZATION2 (VRN2), FERTILIZATION INDEPENDENT ENDOSPERM (FIE), MULTICOPY SUPPRESSOR OF IRA1 (MSI1) and SWINGER (SWN); (4) the plant homeodomain (PHD) proteins VERNALIZATION INSENSITIVE 3 (VIN3), VERNALIZATION 5 (VRN5) and VIN3-LIKE2. The repression of *FLC* involves switching chromatin to a repressed state. The PHD–PRC2 complex acts to deposit the repressive epigenetic mark H3K27me3 across the *FLC* locus, which provides maintenance of *FLC* repression after the return to warm conditions. *FLC* repression proceeds progressively via the cell-autonomous mechanism, and a stable down-regulation of *FLC* is achieved after the prolonged cold exposure, which occurs during winter [6,67,68,69,70,71,72,73,74,75,76].

The mechanisms of *FLC* silencing and epigenetic memory in *Arabidopsis* have been widely studied and summarized in the recent reviews [3,77,78].

**Figure 1:**
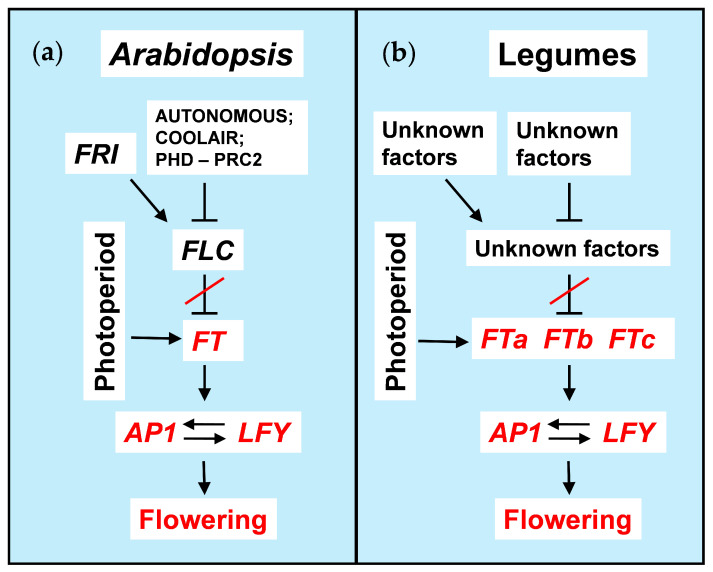
Flowering promotion by vernalization. (**a**) A generalized regulatory mechanism of cold-induced flowering promotion in *Arabidopsis*, (**b**) a putative regulatory network in legumes. Arrows and T-bars show positive and negative regulatory interactions respectively. Activation state of the core components is shown with the red color. ‘Autonomous’ and ‘Photoperiod’ denote the corresponding pathways in *A. thaliana*. *FTa*, *FTb* and *FTc* are three subclades of *FT* orthologs in legumes.

Following the release of repression by FLC, the *FT* and *SOC1* genes become activated by the photoperiodic pathway, leading to flowering promotion [79,80,81,82]. A generalized mechanism of the cold-induced flowering transition in *Arabidopsis* is shown in Figure 1a.

Extensive research revealed that the flowering-related genes are largely conserved between *Arabidopsis* and legumes [42,44,45,49,53,54,55,83,84,85,86,87,88,89,90,91,92,93,94,95,96,97]. However, the legumes generally lack any *FLC* orthologs, except for soybean, a vernalization-independent species, which retained one *FLC* copy in its paleopolyploid genome [42,98,99].

## 3. *FT* Genes in Legumes

Despite the absence of *FLC* orthologs, the legume *FT* genes retain the role of major vernalization signal integrators (Table 2).

Legumes have three subclades of *FT* genes (*FTa, FTb and FTc*) with five *Arabidopsis FT* orthologs detected in pea *Pisum sativum*, and six orthologs in *Medicago trancatula* and *Lens culinaris* (Table 1) [44,45,53,55,56,100]. Unlike pea and *Medicago*, chickpea *Cicer arietinum* has a single *FTb* gene, resulting in a family of five genes (Table 1) [54].

Similarly to the above described legume species, faba bean *Vicia faba* presumably has five or six *FT* genes, however, their number and identity are yet to be determined [57].

The narrow-leafed lupin *Lupinus angustifolius* is a member of the genistoid clade, which is the most basal clade of papilionoid legumes [45,101]. In *Lupinus angustifolius*, the *FTb* subclade is completely missing, while there are two *FTa* genes and two *FTc* genes (Table 1). Phylogenetic analysis revealed that lupin *FTa1* and *FTa2* genes correspond to the *Medicago FTa3* subclade [45]. Lupin *FTa* and *FTc* genes presumably originated from single copies by lineage-specific duplication [90].

Interestingly, the members of different *FT* subclades in pea and *Medicago* showed distinct patterns of regulation with respect to environmental cues and tissue specificity. For example, *FTa* and *FTb* genes are expressed in leaves, while *FTc* genes function in the shoot apex and may contribute to the integration of *FTa* and *FTb* signals [43,44,53]. Moreover, several studies point to the existence of cross-regulation between different members of the *FT* family [44,53]. This demonstrates the complexity of regulatory networks involving various *FT* family members in legumes, including those implicated in the vernalization response (Figure 1).

## 4. A Role of the Legume *FT* Genes in Vernalization Response

### 4.1. Medicago

The study by Laurie et al. provided the first clue on the role of *FT* family genes in the vernalization response in legumes [44]. It was shown that *FTa1* mutants in *Medicago trancatula* are late flowering and have the reduced ability of vernalization response [44,100]. Remarkably, despite vernalization insensitivity, the *MtFTa1* mutants retained the photoperiod response [44].

The experiments on *MtFTa1* overexpression showed the early flowering phenotype in unvernalized plants, resembling that of wild type plants after vernalization. Interestingly, the expression of the *M. trancatula FTc* gene was significantly reduced in *MtFTa1* mutants, suggesting that the *MtFTc* gene might be downstream of *MtFTa* [44].

An insertion of *Tnt1*, a long terminal repeat (LTR) retrotransposon from tobacco, into *FTa1* 3rd intron or in 3′ region resulted in the early flowering phenotype (“spring” mutants), similar to that after *FTa1* overexpression [46,48]. This insertion eliminated vernalization response, but resulted in a strong photoperiod sensitivity. These data suggest that the *MtFTa1* gene may have evolved separate mechanisms of response to vernalization and photoperiod.

Several transcription factors, such as *SOC1* and *FRUITFULL (FUL)* showed elevated expression in spring mutants, which is consistent with their downstream position relative to *FT* signaling in *Arabidopsis* [46,48,100]. The *Arabidopsis FUL* gene encodes a MADS-box protein and acts as a flowering-time and meristem-identity gene, closely related to *AP1* and *CAULIFLOWER (CAL)* [102]. *FUL* is involved in an up-regulation of the *LFY* gene. The *MtSOC1* genes demonstrated up-regulation by both vernalization and photoperiod, which was reduced in *FTa1* mutants, providing evidence that these genes are downstream *FTa1* [49].

An essential role of *FTa1* in *Medicago* was confirmed by a recent RNAseq assay showing a wide range of genes differentially regulated by mutation of *MtFTa1* [96]. Upon floral transition, the *MtFTa1* targets included genes from photoperiod, gibberellin-related and age-related pathways.

In *Arabidopsis*, the FT protein acts in complex with the basic domain/leucine zipper (bZIP) transcription factor *FLOWERING LOCUS D (FD)*, and the model of FT-FD interaction is conserved in different plant species [89,103,104,105,106,107]. The FT-FD heterodimer is responsible for activation of *AP1* [25,106], *FUL* and *SEPALLATA3 (SEP3)* [108]. Interestingly, the mutation in the *MtFDa* gene did not abolish vernalization responsiveness, unlike the mutation in *MtFTa*. Nevertheless, the double mutation of *MtFDa* and *MtFTa1* completely blocked floral transition pointing to the complementary roles of these two genes [96].

### 4.2. Narrow-Leafed Lupin

Studies on the narrow-leafed lupin (*Lupinus angustifolius*) revealed that of all *FT* genes, only *LanFTc1* shows strong vernalization response [45]. The expression of this gene was repressed in the unvernalized conditions, but possessed strong induction following cold treatment.

This is the first case of the *FTc* subclade gene involvement in vernalization response [45]. Of all *FT* subclades, *FTc* genes are the most divergent and differ from most other *FT* genes by substitution of several conserved residues [109].

It has been shown that several natural mutations (*Ku, Jul* and *Pal*) provide vernalization independence in *L. angustifolius*. All these mutations are located in the promoter region of the *LanFTc1* gene [45,47,90,110].

*Ku* and *Jul* are naturally occurring dominant mutations, independently discovered in cultivars “Borre” and “Krasnolistny” in 1960s, which determine vernalization insensitivity and early flowering of the cultivated lupin [110,111,112]. *Ku* and *Jul* are deletions within the *LanFTc1* promoter region, spanning 1423 and 5162 bp respectively. Another 1208 bp deletion, partially overlapping with *Ku* and *Jul*, has been revealed in the wild germplasm from Palestine, and termed *Pal* mutation. *Pal* is associated with a partial vernalization responsiveness and slightly delayed flowering [110].

The analysis of *LanFTc1* expression in the three deletion variants confirmed their importance for flowering time regulation via the vernalization pathway. Remarkably, *Ku* and *Jul* deletions appeared to be functionally equivalent, resulting in the similar expression profile of *LanFTc1* gene. Therefore, it has been suggested that major regulatory elements responsible for *LanFTc1* vernalization sensitivity, confer to a region of *Ku* deletion, namely, the 1423 bp sequence [110]. The functional activity of 1208 bp *Pal* deletion presumably refines this major regulatory region.

An effect of *Ku, Jul* and *Pal* mutations in the narrow-leafed lupin is very similar to that of “spring” mutations in *Medicago*, suggesting a common mechanism of vernalization response by de-repression of *FT* genes [46]. In both cases, current research provides evidence about the genomic regions, containing important binding sites, which enable transcriptional repression of the *FT* genes in wild type. However, in both *Medicago* and lupin, it is unknown which elements within these regions are responsible for the *FT* repression in the absence of vernalization and its de-repression after vernalization [45,110].

The promoter region of the *L. angustifolius LanFTc1* gene contains putative binding sites for the homologues of many transcription factors regulating *FT* expression in *Arabidopsis* [45,110]. This region has been divided into two zones. The deletion of the first zone $C_{H}$ provided a high level of *LanFTc1* expression, while the deletion of the second zone $C_{M}$ resulted in the moderately high expression levels. However, the function of these sequences in wild type remains unclear [110]. In *Arabidopsis*, the *FT* promoter contains four major blocks, including the ID block with two insertions/deletions (indels) affecting promoter efficiency [12,113]. A position of the ID block within the *Arabidopsis FT* promoter is similar to a position of the 1423 bp *Ku* deletion in *L. angustifolius LanFTc1* promoter. However, the *Ku* region showed no sequence conservation with the ID block [90,110].

Besides *LanFTc1*, recent studies have shown the involvement of a number of novel candidate genes in the vernalization response [47,111]. The expression profiles of these genes were examined for vernalization responsiveness in three accessions, carrying domesticated allele *Ku*, intermediate allele *Pal*, and wild allele *ku* [47].

*LanAGL8 (AGAMOUS-LIKE 8)* is an *L. angustifolius* ortholog of the *A. thaliana FUL* gene [115,116,117]. In *L. angustifolius*, the expression pattern and vernalization responsiveness of *LanAGL8* were very similar to those of *LanFTc1*. The authors suggested that *LanAGL8* could act downstream of *LanFTc1*. As described above, an expression of *FUL* in *Medicago* also pointed to its downstream position relative to *MtFTa1* gene [46,48].

*LanFD*, the *L. angustifolius* ortholog of the *Arabidopsis FD* gene, showed vernalization responsiveness, which varied between early and late flowering genotypes. Since the protein product of *FD gene* in *Arabidopsis* acts in a complex with the FT protein [103], a variation in *FD* expression could modulate an effect of the *FT* signal [47].

A recent study provided transcriptomic evidence on the role of *LanCRLK1* and *LanUGT85A2* genes, acting in C-repeat binding factor (CBF) cold responsiveness and UDP-glycosyltransferase pathways, in the *LanFTc1*-mediated vernalization response [114]. Nevertheless, the analysis of *LanCRLK1* and *LanUGT85A2* expression profiles did not provide convincing evidence for the role of these genes in the vernalization response of *L. angustifolius* [47].

### 4.3. Yellow Lupin

Despite the similar domestication history of narrow-leafed lupin and yellow lupin, (*Lupinus luteus*), the genetics of domestication traits have been studied much more intensively in the narrow-leafed lupin [50,118,119]. The studies on QTL mapping of domestication syndrome and flowering time traits in *L. luteus* were first published in 2020 [50,120].

In these studies, the vernalization response QTL of *L. luteus* was mapped to the same syntenic position, as the *Ku* locus of *L. angustifolius*. This locus harbors the orthologous *FTc* gene, which presumably has similar functions as *L. angustifolius LanFTc1* [50,120].

### 4.4. White Lupin

Remarkably, unlike narrow-leafed and yellow lupin, in the white lupin *Lupinus albus* the vernalization response was revealed as a continuous trait suggesting polygenic regulation [52,121]. Despite the involvement of different number of genes (single genes vs several QTLs), the genomes of white and narrow-leafed lupin showed high collinearity [121].

Genotyping with PCR-based markers highlighted major candidates for the vernalization response and early flowering QTLs [51,52,121]. The QTL analyses revealed significant correlations between time to flowering and polymorphism in markers anchored in the sequences of regulatory genes. These genes are slightly divergent between two studies involving mapping population and germplasm collection [51,52]. and include *L. albus* orthologs of *FTa1, FTc1, SEP3, EARLY FLOWER 1 (ELF1), FLOWERING LOCUS D (FLD), FRIGIDA (FRI) CONSTANS (CO), FY, MOTHER OF FT AND TFL1 (MFT), PHYTO-CHROME INTERACTING FACTOR4 (PIF4), SKI-INTERACTING PROTEIN 1 (SKIP1)*, and *VERNALIZATION INDEPENDENCE 3 (VIP3)* [51,52]. Remarkably, both studies highlighted a possible role of the *FRI* gene, a major component of the vernalization pathway, which is responsible for activation of *FLC* expression (see above) [65]. In *Arabidopsis*, an allelic variation of *FRI* accounts for the majority of natural variation in flowering time [122,123].

Overall, these studies highlight a complex nature of flowering control in white lupin, with candidate genes dispersed among numerous loci. This contrasts with rather simple mechanisms of cold-induced flowering in *L. angustifolius* and *L. luteus*.

### 4.5. Garden Pea

In the garden pea *Pisum sativum, the FTa1* gene, corresponding to the *GIGAS* locus described in earlier studies [124], plays a major role in flowering induction [53]. The *gigas* mutants demonstrated late flowering in all photoperiodic conditions tested, nevertheless, with an unambiguous effect of vernalization on flowering time [124]. However, such an effect was observed in the specific tissues, suggesting that *FTa1/GIGAS* may respond to vernalization in a tissue-specific manner [53,124,125]. Acquisition of more molecular data is required to analyze mechanisms of vernalization-induced flowering in pea.

### 4.6. Chickpea

In chickpea *Cicer arietinum*, a major QTL providing 55% of the phenotypic variation for vernalization response trait has been identified on the linkage group 3 (LG3) of the chickpea genetic map [126]. LG3 on chromosome 3 was earlier reported to harbor flowering time genes by many authors [127,128,129,130,131]. Thus, LG3 harbors several QTLs for flowering time and vernalization response in chickpea [126].

A recent study detected a strong genetic association between early flowering and a cluster of *FT* genes on chromosome 3, comprising *FTa1, FTa2* and *FTc* [54]. This cluster has been located to the same genomic region as the QTL for vernalization response [126]. Nevertheless, it is unknown, which of these *FT* genes might be responsible for the effects on vernalization response.

### 4.7. Lentil

A recent QTL analysis revealed that the *DTF6a* locus on lentil (*Lens culinaris*) chromosome 6 strongly contributes to early flowering of the Indian landrace ILL 2601 [55].

The detailed inspection of the *DTF6a* region revealed that it corresponds to the syntenic regions on *Medicago* chromosome 7, pea chromosome 5 and chickpea chromosome 3. This region harbors a conserved cluster of *FT* genes, including two *FTa* genes (*FTa1* and *FTa2*) and an *FTc* gene, located adjacent or nearby [44,53,54,132].

Further analysis showed that *DTF6a* deletion is associated with elevated expression of *FTa1* and *FTa2* genes. The sequences of *FTa1-FTa2* cluster were compared between the early flowering lentil accession ILL 2601 and the late flowering accession ILL 5588. There were no differences in the coding regions of either gene, but the comparison of non-coding regions identified 136 nucleotide polymorphisms (SNPs) and 25 indels distinguishing two accessions. The most substantial difference was the 7441 bp deletion, comprising most of the *FTa1–FTa2* intergenic region in the early ILL 2601 accession [55].

The analysis of vernalization response showed that the late ILL 5588 accession was vernalization-sensitive under long and short photoperiods. On the contrary, ILL 2601 did not show any vernalization-induced acceleration of flowering.

The results suggested that *FTa1* (*LcFTa1*) is the most prominent candidate for the early flowering and vernalization insensitivity of the early ILL 2601 accession [55]. As described above, *FTa1* is responsible for early flowering in *Medicago* and pea [44,53]. A large 7441 bp indel detected in the *FTa1–FTa2* intergenic of the early ILL 2601 accession might include regulatory elements required for *LcFTa1* repression [55]. In this case a mechanism, which confers early flowering and vernalization independence in lentil, should resemble the above described *Ku* mutations in the promoter region of the *FTc1* gene in *L. angustifolius* [45] and transposon insertions in the third intron or 3′ untranslated region of the *FTa1* gene in *M. trancatula* [46].

These results were confirmed in another recent study, considering differences in flowering time between *L. culinaris* and its wild ancestor *L. orientalis* [56]. This study detected QTL, colocalizing with *DTF6a* locus, and revealed differential regulation of *LcFTa1* gene within this QTL [56].

### 4.8. Faba Bean

In faba bean *Vicia faba*, the candidate gene mapping and the quantitative trait loci (QTL) analysis revealed the cluster of *FT* loci close to the most conserved flowering time QTL on chromosome 5 in a region syntenic with a section of *Medicago* chromosome 7 containing *FTa* and *FTc* genes [58]. However, the authors failed to map individual *FT* genes within this region due to high sequence conservation [58].

In another recent study, the single-molecule, real-time (SMRT) sequencing revealed 50 flowering-related genes that could be associated with vernalization. The vernalization-responsive transcripts showed significant up-regulation in the cold-treated samples [57].

The expression dynamics of one candidate gene, an ortholog of *SOC1*, was examined by RT-PCR. Phylogenetic analysis demonstrated that *VfSOC1* is most similar to *MtSOC1c* in *Medicago* (86.5% identity). Under a low temperature treatment, the levels of *VfSOC1* in a leaf showed elevation on day 7 and steadily increased thereafter. The ectopic expression of *VfSOC1* in *Arabidopsis* could promote earlier flowering. These results point to the important role of *VfSOC1* in vernalization response of faba bean [57].

## 5. Candidates for the Vernalization-Repressed Repressors in Legumes

The repressors of flowering, down-regulated by cold exposure, have yet to be uncovered in legumes. As a starting point, current research aims to analyze legume orthologs of the *Arabidopsis* genes involved in the regulation of *FLC* locus.

*FRI* is a major gene controlling vernalization response in *Arabidopsis*. *FRI* suppresses flowering mostly through the upregulation of *FLC* [7]. The analysis of *A. thaliana* populations revealed that the allelic variation of *FRI* accounts for approximately 70% of flowering time variation [123]. In the white lupin *L. albus, FRI* has been recently highlighted as a candidate gene underlying the early flowering QTL [51,52].

As described above, PHD proteins VIN3 and VRN5 are the components of PHD–PRC2 complex, required for the stable repression of *FLC* following vernalization in *Arabidopsis* [70,74,133]. Despite their similar function, *VIN3* and *VRN5* genes differ in the expression dynamics and the vernalization responsiveness [133]. There are three copies of each gene in the genome of narrow-leaved lupine *L. angustifolius*. The expression of *LanVIN3* and *LanVRN5* genes was different from that of *Arabidopsis* with significantly higher levels of *LanVIN3* compared to *LanVRN5* [47]. However, the differences in the expression profiles and vernalization sensitivety of the *LanVIN3* and *LanVRN5* genes did not match the observed differences in time to flowering. Therefore, the authors suggested that these genes are unlikely to be involved in the vernalization response [47].

The VRN2 protein is a core component of Polycomb repressive complex 2 (PRC2) in *Arabidopsis*. Phylogenetic analysis revealed the *VRN2*-like genes in some legume species including *Medicago*, pea and lupin [67]. Interestingly, in *Medicago, MtVRN2* gene functions as a repressor of the floral activator *FTa1* [134]. *Mtvrn2* mutants demonstrated early, vernalization-independent flowering, as well as an elevated expression of *FTa1* and its target genes, including *SOC1a*, *FULb* and *AP1*. Thus, in the absence of the *FLC* clade of flowering time repressors in *Medicago, MtVRN2* apparently plays a role in silencing major flowering activators [134].

Nevertheless, the “memory of the cold”, or prolonged activation of flowering after vernalization, is preserved in *M. trancatula. MtFTa1* gene was not expressed in the germinated seedlings after 14 days in the cold. However, its expression started immediately after plants were transferred to warm conditions, and the expression levels increased after 7 and 14 days in the warmth [44]. The mechanisms of such memory of the cold exposure are yet unknown.

## 6. Conclusions

Vernalization is a widespread mechanism of flowering time regulation in annual plants from temperate regions. However, the vernalization pathways appear to have evolved independently in various plant lineages, leading to differences of gene functions and regulatory pathways across groups [1,109]

Since the functions of *VIN3*, *VRN5* and *VRN2* genes in legumes differ from those in *Arabidopsis*, and the *FLC* gene is absent, it is obvious that the cold-induced de-repression of flowering activators proceeds via different mechanisms. There could be several scenarios of vernalization-based *FT* induction in legumes.

For example, in cereals, vernalization involves the down-regulation of the *FT* repressor, which is not orthologous to *FLC* in *Arabidopsis* [135,136]. On the other hand, in the sugar beet *Beta vulgaris*, vernalization represses an *FT* gene (*BvFT1*), which down-regulates another *FT* gene (*BvFT2*) [137].

Since the *MtVRN2* gene in *Medicago* performs a function opposite to that in *Arabidopsis*, namely, it represses *FTa1* instead of activation [134], the latter scenario is quite plausible. There may also be cross-regulatory interactions between different *FT* genes, as it has been suggested that the *Medicago MtFTc* gene acts downstream of *MtFTa1* [44]. This suggests a complex network transmitting the vernalization signal from several *FT* genes to the meristem identity genes *AP1* and *LFY* (Figure 1b). It should be noted that in addition to being activated by the *FT* orthologs, the legume genes *AP1* and *LFY* are regulated by several copies of the *TFL1* gene [53,91,109,138,139]. Future research should explore this regulatory complexity in terms of the vernalization signal propagation.

## Figures and Tables

**Table 1:** The orthologs of *Arabidopsis FLOWERING LOCUS T (FT)* gene in legumes.

Legume Species	*FT* Genes
*Medicago trancatula*	*FTa1*, *FTa2*, *FTa3*, *FTb1*, *FTb2*, *FTc*
*Pisum sativum*	*FTa1*, *FTa2*, *FTb1*, *FTb2*, *FTc*
*Lens culinaris*	*FTa1*, *FTa2*, *FTa3*, *FTb1*, *FTb2*, *FTc*
*Cicer arietinum*	*FTa1, FTa2, FTa3, FTb, FTc*
*Lupinus angustifolius*	*FTa1*, *FTa2*, *FTc1*, *FTc2*

**Table 2:** Major vernalization targets (*FT* and *SOC1* genes) in different legume species.

Legume Species	Targets of Vernalization	References
*Medicago trancatula*	*FTa1, SOC1*	[44,48]
		[46,49]
*Lupinus angustifolius*	*FTc1*	[45]
*Lupinus luteus*	*FTc1*	[50]
*Lupinus albus*	polygenic regulation,	[51]
	including *FTa1* and *FTc1*	[52]
*Pisum sativum*	*FTa1* (?)	[53]
*Cicer arietinum*	*FTa1, FTa2, FTc* (?)	[54]
*Lens culinaris*	*FTa1*, *FTa2*	[55,56]
*Vicia faba*	*FT, SOC1*	[57]
		[58]

## Data Availability

Not applicable.
